# Antenatal depression among pregnant mothers in Afghanistan: A cross-sectional study

**DOI:** 10.1186/s12884-024-06548-2

**Published:** 2024-05-04

**Authors:** Shararah Sarem, Ahmad Neyazi, Abdul Qadim Mohammadi, Mehrab Neyazi, Mozhgan Ahamdi, Nosaibah Razaqi, Sadaf Wali, Shashank Timilsina, Hamida Faizi, Mark D. Griffiths

**Affiliations:** 1 Herat Maternity Hospital, Herat, Afghanistan; 2 Afghanistan Center for Epidemiological Studies, Herat, Afghanistan; 3 Mental Health Ward, Herat Regional Hospital, Herat, Afghanistan; 4 Matrisishu Miteri Hospital, Pokhara, Nepal; 5 Department of Psychology, Nottingham Trent University, Nottingham, UK

**Keywords:** Depression, Prenatal, Pregnant, Women, Afghan

## Abstract

**Background:**

Approximately one in five pregnant women experience antenatal depression globally. The purpose of the present study was to estimate the prevalence of antenatal depression and explore its relationship between various demographic variables, recent sexual engagement, and recent adverse life events among pregnant Afghan women.

**Methods:**

A cross-sectional survey study was carried out between January, 2023 and April 2023 among 460 women aged 15–45 years who were recruited using convenience sampling from Herat province (Afghanistan). Logistic regression models were utilized to explore the relationship between antenatal depression and socio-demographic characteristics among the participants.

**Results:**

The prevalence of antenatal depression symptoms was 78.5%. Multiple regression analysis indicated that antenatal depression was significantly associated with (i) being aged 30–45 years (AOR: 4.216, 95% CI: 1.868–9.515, *p* = .001), (ii) being of low economic status (AOR:2.102, 95% CI: 1.051–4.202, *p* = .036), (iii) not being employed (AOR: 2.445, 95% CI:1.189–5.025, *p* = .015), (iv) not having had sex during the past seven days (AOR: 2.335, 95% CI: 1.427–3.822, *p* = .001), and (v) not experiencing a traumatic event during the past month (AOR:0.263, 95% CI: 0.139–0.495, *p* < .001).

**Conclusion:**

The present study provides insight into the factors associated with the high prevalence of antenatal depression among pregnant Afghan women (e.g., demographic variables, recent adverse life events, and recent sexual engagement). It highlights the urgency of addressing antenatal depression in Afghanistan and provides a foundation for future research and interventions aimed at improving the mental health and well-being of pregnant women in the Afghan context.

## Introduction

Antenatal depression, also known as prenatal depression, is a condition characterized by symptoms such as low mood, loss of interest or pleasure in previously enjoyed activities, fatigue, hopelessness, restlessness, changes in appetite, alterations in sleep habits, difficulty concentrating, and thoughts of self-harm, social isolation, and fear of attachment issues [1]. To diagnose antenatal depression, these symptoms must persist for at least two weeks and represent a change from the individual’s previous level of functioning among pregnant women [1, 2].

Approximately one in five pregnant women experience antenatal depression globally [3]. The prevalence is even higher in lower- and middle-income countries (LMICs), affecting approximately one in four pregnant women [4]. In Afghanistan (where the present study was carried out), one study reported that the prevalence of depression among pregnant women was 60.9% [5]. In comparison, the prevalence of major depressive episodes among the national Afghan population was reported to be 11.7% [6]. However, a very recent study in the Herat region of Afghanistan reported the prevalence of depression among women to be 80.4% [7]. This is similar to a study that reported that the prevalence of psychological distress symptoms was 75% among the national Afghan population [8].

The magnitude of antenatal depression varies across countries, generally displaying lower rates in developed countries like Japan (5.6%), South Korea (8.1%), and the USA (6.1%) [9–11], while higher rates are reported in developing countries such as Nepal (18%), Ethiopia (31.1%), Bangladesh (18%), India (21.98%) and South Africa (ranging from 21 to 47%) [12–18]. In a meta-analysis of 589 studies from low-and-middle income countries, Mitchell et al. reported the pooled prevalence of antenatal depression was 24.7% [19]. In another meta-analysis of 49 studies, Dadi et al. reported the prevalence of antenatal depression was 34.0% in low-income countries (15 studies), and 22.7% in middle income countries [20].

Antenatal depression is often underrecognized and undertreated, partly due to the overlap of its symptoms with those of a normal pregnancy, including sleep changes, appetite alterations, and fatigue [21]. However, failing to identify and address antenatal depression can have significant consequences. Women with antenatal depression face increased risks of preterm birth, low birth weight, and intrauterine growth restriction [22–25]. Moreover, antenatal depression can lead to poor health behaviors, such as noncompliance with antenatal care, an increased risk of substance abuse, cigarette smoking, lack of exercise, and failure to adhere to taking essential prescription medication [26, 27]. The most concerning complication associated with antenatal depression is suicide [28]. In addition to the direct impact on the mother’s health behavior, antenatal depression can also have adverse effects on the fetus through biochemical pathways [29]. Alterations in the serotonin system and hormones of the hypothalamic-pituitary-adrenal axis, particularly corticotropin-releasing hormone (CRH), play a crucial role. Elevated CRH levels due to antenatal depression can lead to preterm birth, low birth weight, neurodevelopmental issues, and behavioral challenges. Understanding these effects is essential for informed prenatal care because it influences both maternal and fetal outcomes [30].

Various risk factors have been identified for antenatal depression, including lack of support from society and family, past history or family history of mental illnesses, lack of employment, childhood abuse, adverse life events, and intimate partner violence [21, 31, 32]. It is also worth noting that the majority of cases of postnatal depression are preceded by antenatal depression [10, 33, 34]. Therefore, recognizing and treating antenatal depression is crucial for early diagnosis and management of postnatal depression, which can otherwise result in neglect and mistreatment of the newborn. Sexual activity can trigger the release of endorphins, dopamine, oxytocin, and other neurotransmitters associated with pleasure and well-being [35]. The psychological aspects of sexual activity, such as intimacy, connection, and self-esteem, can also influence mental health [36]. Positive sexual experiences within a supportive and consensual context may promote feelings of happiness, satisfaction, and emotional well-being, which could serve as protective factors against depression [37]. A study by Liu et al. in Taiwan also indicated that many individuals tend to reduce the frequency of sexual activity during pregnancy [38].

To the best of the authors’ knowledge, no previous study has ever examined the factors associated with antenatal depression in Afghanistan. Therefore, the purpose of the present study was to (i) estimate the prevalence of antenatal depression among pregnant Afghan women, and (ii) explore the relationship between antenatal depression and various demographic variables, recent adverse life events, and recent sexual engagement.

## Methods

### Participants, study design, and procedure

A cross-sectional survey study was conducted, and the data were collected between January 2023 and April 2023. The study participants (*N* = 460) were pregnant females aged 15–45 years who were recruited using a cluster sampling technique from Obstetrics & Gynecology departments of public hospitals in Herat province (Afghanistan). Out of 200 public clinics in Herat province, five clinics and hospitals active in Herat city of Afghanistan were selected to participate. In each cluster, participants were selected using convenience sampling. To recruit the participants, trained data collectors went to relevant departments in public hospitals and clinics in Herat province. They then asked the pregnant women visiting the hospitals and clinics to participate in the study. The women who agreed to participate were then interviewed by the data collectors. The target sample size of participants was determined using the formula N = Zα2P(1 − P)/d2, in which α = 0.05 and Zα = 1.96, and the estimated acceptable margin of error for proportion d was 3.6%. The proportion of women with depression was estimated at 80%, based on the available literature [39]. This resulted in a minimum sample size of 461 participants. Participants were interviewed face-to-face and their answers were recorded by the data collectors. The eligibility criteria to participate in the study were: (i) being female, (ii) being pregnant, (iii) being able to understand the Dari language, and (iv) being able to provide written or verbal informed consent.

### Instruments

The present study utilized a survey comprising two distinct sub-sections to evaluate various aspects related to participants’ socio-demographic characteristics and symptoms of depression. The socio-demographic section of the survey encompassed a range of questions to gather information on key factors such as age, residency, education level (lower education [illiterate or primary school] and higher education [secondary school, high school or university]), husband’s education, economic status (high income [having a monthly family income of more than $100] and low income [having a monthly family income of less than $100]), occupation, husband’s occupation, recent sexual activity (*“How many days has it been since you last had sex?”*), and any traumatic events experienced during the previous month. A traumatic event constituted any self-defined distressing or deeply disturbing incident or experience that the participant may have encountered within the past 30 days. This could include events such as accidents, physical or emotional harm, violence, natural disasters, bereavement of family members or any other situation that significantly impacted them in a negative way. These socio-demographic factors may play a role in understanding the contextual background and potential influences on participants’ mental health.

To assess the presence and severity of depression symptoms among participants, the Persian version of the Edinburgh Postnatal Depression Scale (EPDS) was used [40]. The 10-item scale was specifically designed to assess both postpartum and antenatal depression symptoms [40, 41]. Each item of the EPDS represents a statement related to depressive experiences (e.g., *“I have been so unhappy that I have had difficulty sleeping”*). Participants rate their agreement with each statement, assigning scores ranging from 0 (indicating the absence of the symptom) to 3 (representing the highest level of severity). Consequently, the total scores vary between 0 and 30. To determine the presence of antenatal depression symptoms, a standard cut-off score was implemented. Participants who obtained a score between 0 and 9 were classified as having no depressive symptoms. Conversely, participants scoring above 9 were identified as exhibiting signs of antenatal depression. In the present study, the Cronbach alpha of the EPDS was 0.758, indicating a satisfactory level of internal consistency.

### Analysis

The process of data entry was conducted using *Microsoft Excel 2016*, ensuring accurate and organized recording of the study data. Subsequently, the collected data underwent analysis using *IBM SPSS version 26.0 for Windows*. Descriptive statistics, including frequencies and percentages, were calculated to provide a clear summary of the data distribution and characteristics. To explore the relationships between variables, chi-square tests were employed to assess the associations. This provided insights into potential connections and patterns among the study’s categorical variables. Multiple regression analysis was conducted to investigate the impact of independent socio-demographic factors on the presence of antenatal depression symptoms. The significance level for this analysis was set at *p* < .05.

## Results

Almost four-fifths of participants were aged 15–29 years (79.1%), and almost half the participants were from rural areas (46.5%). Almost nine-tenths of participants reported that their economic status was low (89.6%). Only one-tenth of participants were employed (10.4%). More than half of the participants reported not having sex in the past seven days (54.1%) (Table 1).

**Table 1:** Socio-demographic characteristics of participants (*N* = 460)

Characteristic	Category	Number	(%)
Age group	15–29 years 30–45 years	364 96	79.1 20.9
Residency	Urban Rural	246 214	53.5 46.5
Education	Illiterate Primary school Secondary school High school University	189 108 59 55 49	41.1 23.5 12.8 12.0 10.7
Husband’s education	Illiterate Primary school Secondary school High school University	182 93 61 64 60	39.6 20.2 13.3 13.9 13.0
Economic status	High income Low income	48 412	10.4 89.6
Occupation	Employed Un-employed	49 411	10.7 89.3
Husband’s occupation	Employed Un-employed	182 278	39.6 60.4
Had sex during the past seven days	Yes No	211 249	45.9 54.1
Experienced a traumatic event in the past month	Yes No	154 306	33.5 66.5
Total		460	100.0

Four-fifths of the participants had symptoms of antenatal depression (78.5%). Symptoms of antenatal depression were significantly associated with (i) age (older women were more likely to depressed), (ii) residency (women living in rural areas were more likely to be depressed), (iii) educational level (women with lower education were more likely to be depressed), (iv) husband’s educational level (women whose husbands had lower education level are more likely to be depressed), (v) economic status (women with low income were more likely to be depressed), (vi) occupation (employed women were less likely to be depressed compared to women who are not employed), (vii) husband’s occupation (women whose husbands were unemployed were more likely to be depressed), (viii) having sex in the past seven days (women who had sex during the past seven days during their pregnancy were less likely to be depressed), and (ix) traumatic event (women experiencing a traumatic event in the past month were more likely to be depressed) (Table 2).

**Table 2:** Prevalence of depression and its relationship with socio-demographic characteristics of participants (*N* = 460)

Characteristic	Category	Mental health		*p*-value
		Normal, *N*(%)	Depressed, *N*(%)	
Age group	15–29 years 30–45 years	91 (25.0) 8 (8.3)	273 (75.0) 88 (91.7)	< 0.001
Residency	Urban Rural	63 (25.6) 36 (16.8)	183 (74.4) 178 (83.2)	0.022
Education	Illiterate Primary school Secondary school High school University	26 (13.8) 27 (25.0) 17 (28.8) 16 (29.1) 13 (26.5)	163 (86.2) 81 (75.0) 42 (71.2) 39 (70.9) 36 (73.5)	0.018
Husband’s education	Illiterate Primary school Secondary school High school University	23 (12.6) 21 (22.6) 18 (29.5) 22 (34.4) 15 (25.0)	159 (87.4) 72 (77.4) 43 (70.5) 42 (65.6) 45 (75.0)	0.002
Economic status	High income Low income	18 (37.5) 81 (19.7)	30 (62.5) 331 (80.3)	0.004
Occupation	Employed Un-employed	18 (36.7) 81 (19.7)	31 (63.3) 330 (80.3)	0.006
Husband’s occupation	Employed Un-employed	51 (28.0) 48 (17.3)	131 (72.0) 230 (82.7)	0.006
Had sex during the past seven days	Yes No	63 (29.9) 36 (14.5)	148 (70.1) 213 (85.5)	< 0.001
Experienced a traumatic event in the past month	Yes No	14 (9.1) 85 (27.8)	140 (90.9) 221 (72.2)	< 0.001
Total		99 (21.5)	361 (78.5)	

Multiple logistic regression analysis was run to see which variables predicted antenatal depressive symptoms comprising the following variables: age, residency, economic status, occupation, husband’s occupation, having sex during the past seven days, and experiencing a traumatic event in the past month. Antenatal depression was significantly associated with (i) being aged 30–45 years (AOR: 4.216, 95% CI: 1.868–9.515, *p* = .001), (ii) being of low economic status (AOR:2.102, 95% CI: 1.051–4.202, *p* = .036), (iii) not being employed (AOR: 2.445, 95% CI:1.189–5.025, *p* = .015), (iv) not having had sex during the past seven days (AOR: 2.335, 95% CI: 1.427–3.822, *p* = .001), and (v) not experiencing a traumatic event during the past month (AOR:0.263, 95% CI: 0.139–0.495, *p* < .001) (Table 3).

**Table 3:** Multiple logistic regression analysis of predictors of antenatal depression among participants (*N* = 460)

Variable	AOR [95% CI]	*p*-value
*Age group*		
15–29 years	Reference	0.001
30–45 years	4.216 [1.868, 9.515]	
*Residency*		
Urban	Reference	0.524
Rural	1.182 [0.707, 1.975]	
*Economic status*		
High income	Reference	0.036
Low income	2.102 [1.051, 4.202]	
*Occupation*		
Employed	Reference	0.015
Unemployed	2.445 [1.189, 5.025]	
*Husband’s occupation*		
Employed	Reference	0.293
Unemployed	1.307 [0.793, 2.152]	
*Having sex during the past seven days*		
Yes	Reference	0.001
No	2.335 [1.427, 3.822]	
*Experiencing a traumatic event during the past month*		
Yes	Reference	< 0.001
No	0.263 [0.139, 0.495]	

AOR: Adjusted odds ratio

## Discussion

To the best of the present authors’ knowledge, the present study is the first to investigate the prevalence of antenatal depression among pregnant Afghan women. The study examined the relationship between antenatal depression (using the Persian version of the Edinburgh Postnatal Depression Scale [EPDS]) and various demographic variables, recent adverse life events, and recent sexual engagement among 460 pregnant women. This rate was higher than previous studies on depression in Afghanistan among different population groups which have reported rates of 11.7-67.7% among the general population [8, 42], and 60.9% depression among pregnant women [5], but similar to a study reporting the prevalence of 80.4% depression among Afghan women [7]. This rate was also significantly higher compared to other countries’ studies in South Asia, such as Nepal (18%) [12], Bangladesh (18%) [16], India (21.98%) [17], and South Asian countries in general [27.0%] [20]. This suggests an urgent need to address antenatal depression in Afghanistan.

Multiple logistic analysis demonstrated several significant associations between antenatal depression and the assessed factors. Participants aged 30–45 years were four times more likely to experience antenatal depression than those aged between 15 and 29 years old. This concurs with a study among African American women which reported that older women were five times more likely to have antenatal depression than teen mothers [43]. However, some studies from Saudi Arabia and India have shown younger age to be a risk factor for antenatal depression [44, 45], while one study in Ethiopia found no significant association between antenatal depression and age [14].

In the present study, individuals with low income were twice as likely to experience antenatal depression compared to those with higher incomes. Similarly, participants without an occupation were found to be twice as likely to experience antenatal depression (although the similarity in risk of antenatal depression would likely be expected given that not having an occupation would be associated with low income). Low socioeconomic status and unemployment have been consistently identified as key risk factors for antenatal depression [3, 14, 46–49]. As aforementioned, unemployment can contribute to low socioeconomic status, which, in turn, may lead to concerns about fulfilling the basic needs of the baby.

Individuals who did not engage in sexual activity within the past seven days were twice as likely to experience antenatal depression. The results of this study are consistent with the findings of a longitudinal study in Germany [50] which reported that not engaging in sexual activity during the pregnancy results in depression. Also, another study by Nik-Azin et al. found an association between not having sex during pregnancy and depression [51]. The underlying mechanisms for this association could be due to multiple psychological factors (e.g., physical discomfort, or body image concerns) and requires further investigation [52, 53]. Nik-Azin et al. also reported a weak but significant association between female sexual functioning with the general quality of life and its psychological and environmental sub-dimensions [51]. Other studies have shown that having a good sexual quality of life is associated with quality of life more generally which is likely to be associated with lower levels of depression [54, 55].

The present study also found a significant association between recent adverse life events and antenatal depression. Participants who had experienced a traumatic event within the past month were four times more likely to experience antenatal depression. Studies in South Africa [18], South Korea [56], Finland [57], and Singapore [58] have all reported that adverse events in life are associated with antenatal depression. More generally, in a systematic review Biaggi et al. also reported that adverse events in life was significantly associated with presence of antenatal depression [21]. This finding highlights the impact of adverse experiences on mental health during pregnancy and emphasizes the need for comprehensive support systems for pregnant women, particularly those facing challenging life circumstances.

### Strengths and limitations

The key strength of the present study is the use of a standardized instrument to estimate the prevalence of antenatal depression. An inclusive sampling technique and psychometrically validated instruments were used to minimize the study biases. Moreover, the study was the first ever to examine antenatal depression in Afghanistan and comprised a relatively large sample size of pregnant women. However, there are a number of limitations. The study only included pregnant females attending public medical facilities within the urban area of Herat province, therefore the study lacks generalizability to other pregnant women such as those attending private hospitals or those from other provinces within Afghanistan. The data were systematically gathered from healthcare facilities situated within urban locales. However, it is pertinent to acknowledge the constraints in healthcare services prevalent in rural regions, which necessitate some individuals, particularly women, to seek medical attention in urban-based hospitals. Consequently, this resulted in the inclusion of participants from rural areas within the study cohort, thereby providing a broader demographic representation. Given the study’s cross-sectional design, the establishment of causal relationships between depression and associated variables could not be determined. Also, no data regarding the participants’ pregnancy trimester was collected, therefore the associations between depression and specific trimester could not be examined. Moreover, all data were self-reported, therefore the data were subject to various methods biases. Future research with larger and more representative samples (including longitudinal studies) are needed to overcome the limitations of the present study.

## Conclusion

The present study provided insight concerning the high prevalence of antenatal depression among pregnant Afghan women and its associations with demographic variables, recent adverse life events, and recent sexual activity. It highlights the urgency of addressing antenatal depression in Afghanistan and provides a foundation for future research and interventions aimed at improving the mental health and well-being of pregnant women in this context.

The high prevalence of antenatal depression in Afghanistan, coupled with its significant associations with various demographic variables and recent adverse life events, underscores the importance of the need to address this issue. The study suggests the need for targeted interventions that address the social determinants of mental health such as unemployment and low socioeconomic status. Implementing job creation programs, skills training, microfinance initiatives, social support networks, integrating mental health into maternal health programs, and community education can help reduce unemployment and low socioeconomic status, which may help in lowering antenatal depression among Afghan pregnant women. By addressing these underlying factors, such interventions can effectively prevent and mitigate depression among vulnerable populations. The findings of the present study provide valuable insights into the challenges faced by pregnant Afghan women and can inform healthcare professionals, policymakers, and researchers in developing appropriate interventions. Early detection and management of antenatal depression are crucial to prevent adverse outcomes for both the mother and the newborn.

### Ethical considerations

The present study received ethical approval from the Afghanistan Center for Epidemiological Studies - Ethical Committee (reference number #23.1.002), granted on January 1, 2023. Prior to the commencement of the study, participants were provided with a detailed description of the research, ensuring transparency and clarity regarding its purpose, procedures, and potential risks or benefits. Informed consent, either in written or verbal form, was obtained from all participants, signifying their voluntary agreement to participate in the study. Informed consent was obtained from legal guardian of the participants aged less than 18 years old. The data collectors underwent training sessions focusing on data collection techniques. However, it is important to note that the survey responses were not evaluated or scored during the interviews. Consequently, the data collectors were unaware of participants’ depression status. However, they were instructed to promptly refer any participants expressing suicidal ideation to the Department of Mental Health at Herat Regional Hospital. Participants were explicitly informed of their right to withdraw from the study at any stage, without facing any negative consequences or repercussions. This ensured that participants maintained full autonomy and control over their involvement, respecting their rights and well-being throughout the research process.

## Data Availability

All data relevant to the study are available from corresponding author upon reasonable request.
